# Clinical and demographic characteristics associated with nail involvement in alopecia areata: A cross‐sectional study of 197 patients

**DOI:** 10.1002/hsr2.2020

**Published:** 2024-04-01

**Authors:** Safoura Shakoei, Golnoosh Seifi, Farbod Ghanami, Narges Ghandi, Shahin Hamzelou, Maryam Nasimi, Ifa Etesami

**Affiliations:** ^1^ Department of Dermatology, Imam Khomeini Hospital Complex Tehran University of Medical Sciences Tehran Iran; ^2^ Department of Dermatology, Razi Hospital, Tehran University of medical Sciences Tehran University of Medical Sciences Tehran Iran

**Keywords:** alopecia areata, nail changes, severity of illness index, young adult

## Abstract

**Background and Aims:**

Alopecia areata (AA) is an immune‐mediated nonscarring alopecia. Nail changes are a common disfiguring feature of AA with an average prevalence of 30%. We aimed to evaluate the frequency of different types of nail changes and determine demographic and clinical associations.

**Methods:**

This cross‐sectional study included 197 AA patients. Demographic and clinical variables including the Severity of Alopecia Tool (SALT) score, type of AA, and nail changes were evaluated.

**Results:**

Among 197 AA patients with a mean age of 28.95 ± 14.45 years, 50.3% were female. Nail changes were detected in 165 patients (83.8%). The most frequent nail abnormalities were pitting (53.3%), linear line (46.7%), and distal notching (26.9%). AA patients with nail abnormalities were significantly younger than patients without nail changes (25.31 ± 14.96 vs. 32.22 ± 9.77 years; *p* < 0.001). Considering age groups, younger children (less than 10 years) were more likely to have nail changes than adults (97.1% vs. 76.5%; *p* < 0.001). The prevalence of linear line (69.6%) and distal notching (46.4%) were significantly higher in the universalis variant compared to other variants (*p* < 0.001). Pitting (54.5%), distal notching (43.9%), and koilonychia (12.1%) were the most common nail changes in severe forms compared to mild‐to‐moderate forms (*p* < 0.009).

**Conclusions:**

Our study revealed that young patients with severe disease are prone to nail abnormalities. Pitting, distal notching, and linear line were the most common nail changes. Of note, koilonychia, leukonychia, and red spots lunula are more expected in more severe AA.

## 1 INTRODUCTION

Alopecia areata (AA) is the most prevalent autoimmune disorder characterized by nonscaring patchy hair loss.^1^, ^2^ AA affects 2% of the global population^3^ and may occur at any age and peaks between the second and fourth decades of life with no sex predominance.^4^, ^5^ The complex interplay between underlying autoimmune etiopathogenesis, genetic susceptibility, and some environmental factors is suggested as the suspected mechanism.^6^, ^7^, ^8^ The predicted poor prognostic factors include the extent of involvement, ophiasis pattern of hair loss, long duration of hair loss, atopic diseases and other autoimmune diseases, a positive family history, and nail involvement.^9^, ^10^

Nail involvement was first associated with AA in 1898^11^ and has been reported in 7%–66% of AA patients. However, its prevalence is probably underestimated. Nail changes may be asymptomatic and therefore often overlooked in physical examination. But pain, functional problems, and a decrease in health and quality of life due to cosmetic disfigurement have also been documented. Nail changes occur more commonly in children and patients with severe variants of AA like alopecia universalis (AU) and alopecia totalis (AT). AA‐associated nail changes may occur at any time in the clinical course.^12^ The exact pathogenic mechanism of nail changes in AA is unknown, but it has been proposed that nails are affected by the same type of inflammatory cells that target hair follicles. Because the nails and hair follicles are similar in structure and growth.^13^ Histopathological observations of nail changes confirm abnormalities in matrix keratinization. Therefore, the proximal nail matrix is predominantly involved compared to the nail bed which leads to clinical presentations like pitting, trachyonychia, onychomadesis, and nail thinning with or without koilonychia.^2^

Data regarding nail abnormalities in patients with AA are sparse. We aimed to evaluate nail involvement and its association with demographic and clinical characteristics in patients with AA.

## 2 MATERIALS AND METHODS

A cross‐sectional study was conducted on a sample of 197 confirmed AA patients between 2021 and 2022, referred to dermatology departments of Imam Khomeini and Razi hospitals, Tehran University of medical sciences, Tehran, Iran. The included AA patients were then evaluated by two expert dermatologists for the presence of nail findings. Nails were also carefully examined for subungal hyperchratosis and onychodytrophy, and KOH mount and culture for dermatophytes were performed when necessary to rule out onychomychosis. Nail changes were attributed to AA after exclusion of other cutaneous or systemic diseases, including psoriasis. The inclusion criteria were all the confirmed AA patients referred to dermatology departments of Imam Khomeini and Razi hospitals between 2021 and 2022 regardless of age, gender, and disease duration. The exclusion criteria were as follows: 1. Recent history of nail trauma; 2. Acute or chronic nail diseases (except AA‐associated) in the past 3 months; 3. No consent to participate in research.

Demographic data, family history, associated diseases such as other autoimmune diseases, and atopic dermatitis were recorded. To obtain better analytic data, we defined three age groups: ≤10, 11–18, and >18 years old. The clinical type of lesions was categorized as patchy AA, ophiasis pattern, AT, and AU. The mean duration of disease, treatment history, and severity of AA was obtained from the review of medical records. Disease duration was classified as ≤3, 3–9, ≥9 years. The severity of AA was determined by the scoring system of AA called the Severity of Alopecia Tool (SALT). The SALT score is a global severity score in percentage based on the extent and density of visual hair loss in four views of the scalp.^14^ SALT categories were described as follows: no hair or limited = 0%–20%; moderate = 21%–49%; severe and very severe = 50%–100%.^15^ Gender, age, AA variant, disease duration, and SALT score were compared between AA patients with and without nail changes. Moreover, the association between each type of nail changes and these variables was evaluated.

Data analysis was performed using the IBM SPSS version 26 (IBM SPSS Statistics for Windows, Version 26.0., IBM Corp.). All categorical variables were reported as frequencies or percentages, and continuous variables were summarized using mean and standard deviation. The *χ* ^2^ and independent *t*‐test were applied to analyze the categorical variables as appropriate. A *p‐*value of <0.05 was considered statistically significant. The present study was approved by the ethics committee of the Tehran University of Medical Sciences. Verbal and written informed consents were obtained from each participant or their parents in case of underage by the interviewer before conducting the interview; it included an explanation regarding the method and the aims of the research. Participation in this study had no complications or additional costs for the patients.

## 3 RESULT

### 3.1 General demographic and clinical characteristics

Of the total 197 patients, the mean age was 26.43 ± 14.45 years old (range from 2 to 72 years). The mean disease duration and SALT score were 7.82 ± 7.65 years and 43.02 ± 33.79%, respectively. Family history of AA was positive in 35 (17.8%) patients. Five (2.5%) patients had autoimmune diseases including Hashimoto thyroiditis (two cases), multiple sclerosis (one case), type 1 diabetes (one case), and systemic lupus erythematosus (one case). One (0.5%) patient had atopic dermatitis. Regarding AA variants, 111 (56.3%) patients had patchy AA, 56 (28.4%) cases had AU, 20 patients (10.2%), and 10 (5.1%) cases had AT and ophiasis AA, respectively. Among all cases, 95 (48.2%) patients were taking tofacitinib, while 40 (20.3%) patients were not under treatment. Diphencyprone, cignolin, and prednisolone were the other drugs taken by 38 (19%), 20 (10%), and 4 (2%) patients, respectively.

In our study, 165 (83.8%) patients had at least one nail abnormality (49 patients [29.9%] had one nail change and the remaining more than one). The most frequent nail abnormalities included pitting in 105 (53.3%), linear line in 92 (46.7%), and distal notching in 53 (26.9%) cases. Beau's lines and onychorrhexis were rare findings. The complete details of nail changes are listed in Supporting Information: Table 1.

### 3.2 Comparison between AA patients with and without nail findings

As shown in Table 1, AA patients with nail abnormalities were significantly younger than patients with no nail changes (*p* < 0.001). Patients with nail changes had more frequently severe AA variants (AT and AU) and higher SALT scores, however, the difference was not statically significant (*p* < 0.18).

**Table 1:** Characteristics of patients with and without nail changes.

Variable	Patients with nail findings (*n* = 165)	Patients without nail findings (*n* = 32)	*p* Value
Sex, *n* (%)			
Male	85 (51.5)	13 (40.6)	0.259
Female	80 (48.4)	19 (59.3)	
Age (years), mean ± SD	25.31 ± 14.96	32.22 ± 9.77	0.000*
Type of AA, *n* (%)			
Patchy	87 (52.7)	24 (75)	0.092
Ophiasis	8 (4.8)	2 (6.25)	
Totalis	18 (10.9)	2 (6.25)	
Universalis	52 (31.5)	4 (12.5)	
Positive family history of AA, *n* (%)	29 (17.5)	6 (18.7)	0.874
Comorbidities, *n* (%)			
Atopic dermatitis	1 (0.6)	0	0.659
Other autoimmune diseases	5 (1.8)	0	0.319
Disease duration (years), mean ± SD	7.56 ± 7.7	9.18 ± 7.31	0.832
SALT score, percentage ± SD	45 ± 33.74	30 ± 31.45	0.181

Abbreviations: AA, alopecia areata; SALT, Severity of Alopecia Tool.

*Significant at *p* < 0.05.

### 3.3 Associations between nail changes and demographic and clinical variables

**Figure 1:**
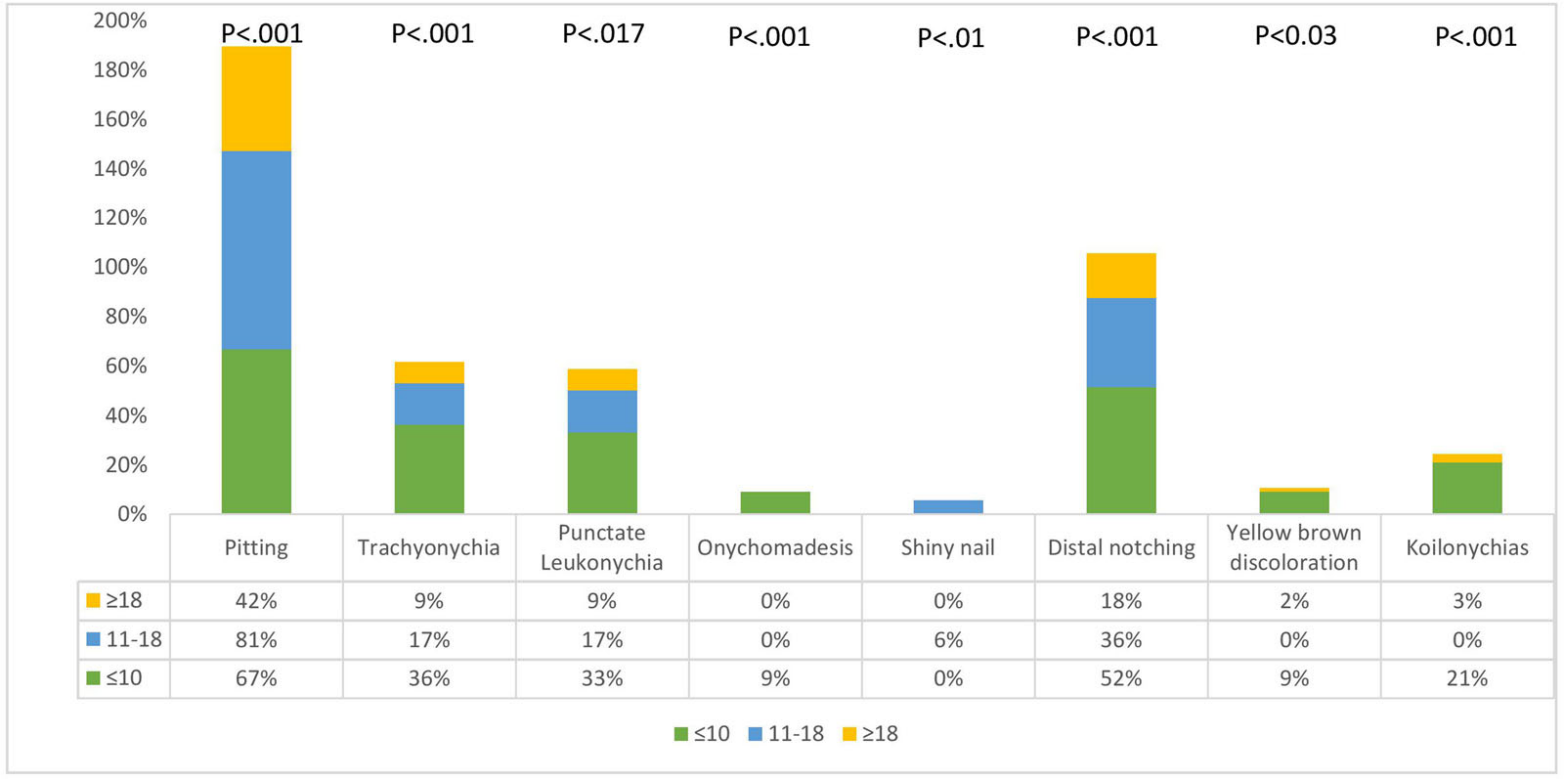
Frequency distribution of some nail changes based the age groups.

The frequency of nail changes was significantly associated with age (*p* < 0.001).Considering the age groups, total nail changes were seen in a higher proportion of children less than 18 years old (67 out of 69; 97.1%) compared to adults (98 out of 128; 76.6%) (*p* < 0.001). This was particularly true for pitting (73.9% vs. 42.2%), distal notching (43.4% vs. 18%), punctate leukonychia (24.6% vs. 12.5%), and trachyonychia (26% vs. 8.6%) (*p* < 0.017). The yellow‐brown discoloration was more commonly detected in children under 10 years old (*n* = 3; 9.1%) than in older patients (*n* = 2; 1.6%) (*p* < 0.03). Details and some age‐specific nail changes are demonstrated in Figure 1 and Supporting Information: Table 2.

The prevalence of nail changes was not significantly different between men and women (*p* < 0.26). Nevertheless, trachyonychia (*n* = 22; 22.4% vs. *n* = 7; 7.1%), linear line (*n* = 56; 57.1% vs. *n* = 36; 36.4%), and distal notching (*n* = 34; 34.7% vs. *n* = 19; 19.2%) were significantly more common in men (*p* < 0.01); and brittle nails were frequently in women detected (*n* = 9; 9.1% vs. *n* = 1; 1%) (*p* < 0.01).

Pitting (*n* = 41; 60.3%), distal notching (*n* = 23; 33.8%), and trachyonychia (*n* = 16; 23.5%) were significantly more commonly observed in patients with a duration of disease less than 3 years (*p* < 0.03).

Linear line (*n* = 39; 69.6% vs. *n* = 38; 34.2%) and distal notching (*n* = 26; 46.4% vs. *n* = 20; 18%) were the most common changes in the universalis variant and the least changes in patchy form (*p* < 0.001). Leukonychia in ophiasis (*n* = 1; 10%) (*p* < 0.02) and koilonychia (*n* = 4; 20%) in totalis variant (*p* < 0.01) were the frequent changes.

Data regarding the most common nail changes is provided in Table 2. Additionally, the complete list of the aforementioned associations is illustrated in Supporting Information: Table 1.

**Table 2:** Associations between the most common nail abnormalities and AA variants.

Nail changes	Frequency, *N* (%)	Age (mean)	SALT score (mean)	AA variant; *N* (%)				
				Patchy (*n* = 111)	Ophiasis (*n* = 10)	AT (*n* = 20)	AU (*n* = 56)	*p* Value
Total nail changes	374 (83.8)	25.31	45.55	87 (78.4)	8 (80)	18 (90)	52 (92.9)	0.092
Pitting	105 (53.3)	22.99	45.79	55 (50.5)	6 (60)	12 (60)	31 (55.4)	0.802
Linear line	92 (46.7)	28.35	50.98	38 (34.2)	5 (50)	10 (50)	39 (69.6)	0.000*
Distal notching	53 (26.9)	22.51	59.08	20 (18)	2 (20)	5 (25)	26 (46.4)	0.001*
Leukonychia	35 (17.75)	21	41	0	1 (10)	0	1 (1.8)	0.021*
Punctate Leukonychia		20.88	51.70	15 (13.5)	2 (20)	5 (25)	11 (19.6)	0.528
Trachyonychia	29 (14.72)	18.69	55.03	11 (9.9)	1 (10)	6 (30)	11 (19.6)	0.071

Abbreviations: AA, alopecia areata; AT, alopecia totalis; AU, alopecia universalis; SALT, Severity of Alopecia Tool.

*Significant at *p* < 0.05.

**Figure 2:**
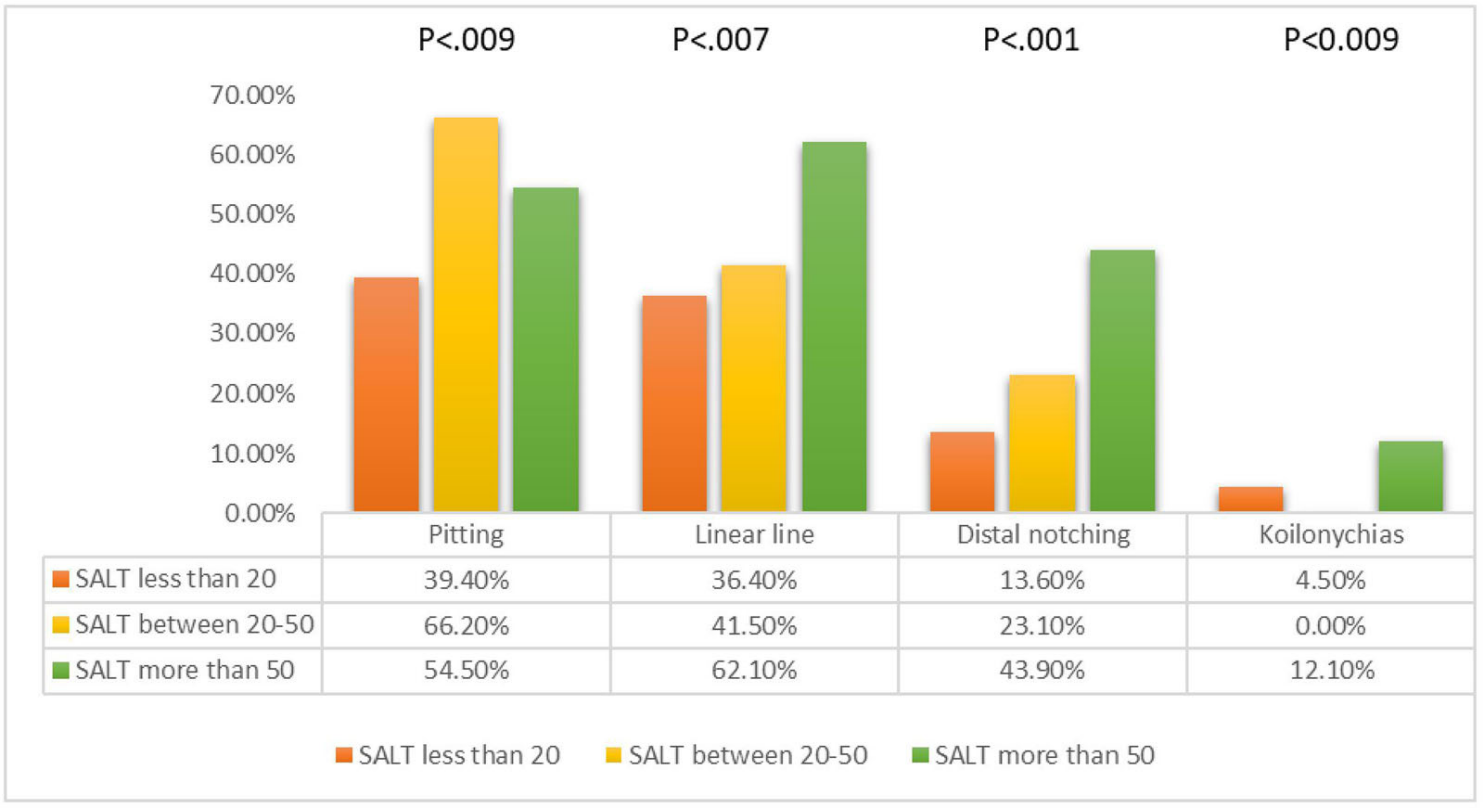
Associations between disease severity based on Severity of Alopecia Tool (SALT) score and nail changes.

The prevalence of nail changes was significantly associated with disease severity (*p* < 0.03). Pitting (*n* = 36; 54.5%), distal notching (*n* = 29; 43.9%), and koilonychia (*n* = 8; 12.1%) were the highest changes in severe forms compared to mild to moderate forms (*p* < 0.009). (Figure 2; Supporting Information: Table 2).

Due to the rarity of atopic dermatitis and other autoimmune diseases, we could not evaluate their relationship with nail changes.

## 4 DISCUSSION

AA is the most prevalent autoimmune disorder characterized by nonscaring patchy hair loss. Nail involvement has been reported in 7%–66% of AA patients and it has been proposed as one of the poor prognostic factors.^12^ However, its prevalence is probably underestimated and overlooked in physical examination. We evaluated nail involvement and its association with demographic and clinical characteristics in patients with AA. Our patients were on average young AA patients with almost 8 years of AA history and moderate to severe disease involvement. We found positive family history of AA in 17.8% of our patients. The most common AA variant was the patchy pattern and then universalis, and tofacitinib was the most commonly prescribed drug. More than 80% of our AA patients had at least one nail abnormality; pitting, linear line, and distal notching were among the most frequent nail findings. On average AA patients with nail abnormalities were younger than patients with no nail changes and had more severe disease involvement. Moreover, some nail abnormalities were specifically associated with gender like brittle nails, trachyonychia, and linear line.

The role of human leukocyte antigen and family history has been suggested by several studies.^16^, ^17^, ^18^ Arousse et al.^19^ showed positive family history in 22.1% of AA patients and proposed thyroid disease as the most common autoimmune comorbidity (12.7%). This is in agreement with our results which detected AA family history in 17.8% and autoimmune comorbidities in 2.5% of the patients including Hashimoto thyroiditis. Our results showed that positive family history of AA is more common than autoimmune comorbidities.

In our study, almost 84% of the patients had at least one nail abnormality. Yesudian et al.^20^ in a review study had shown that about 30% of AA patients (range 7%–66%) had nail involvement. A higher prevalence of nail abnormalities in our patients might be due to the large proportion of patients with high SALT scores and severe AA; the mean SALT score was almost 43%. Of note, in our study either severe AA variants (totalis and universalis) or higher SALT scores were considered as severe AA, however, the majority of the researchers who studied AA‐associated nail changes did not report the severity based on the SALT scoring system. The most prevalent AA patterns were patchy (56.3%) and universalis (28.4%). This finding supports previous studies reporting patchy (49.5%) and universalis (27.5%) patterns as the most common AA variants.^19^ The most frequent nail abnormalities included pitting and linear lines in almost half of the patients. These results match those observed in earlier studies. Trachyonychia was another common AA‐associated nail change reported in the literature.^3^, ^4^, ^12^, ^21^, ^22^, ^23^, ^24^, ^25^, ^26^ These results support the fact that the proximal nail matrix is predominantly affected by AA than the distal matrix and nail bed involvement.^27^ To have a better understanding of the frequency of nail changes, we have used a table from a review study and compared the results (Table 3).^12^

**Table 3:** Frequency of nail abnormalities, by study.

	Tosti et al.^31^ (children) (*N* = 126)	Sharma et al.^23^ (children) (*N* = 841)	Sharma et al.^11^ (adults) (*N* = 761)	Sharma et al.^11^ (children) (*N* = 239)	Tan et al.^26^ (*N* = 219)	Gandhi et al.^24^ (*N* = 100)	Kasumagic‐Halilovic and Prohic^13^ (*N* = 200)	Our study (adults) (*N* = 128)	Our study (children) (*N* = 69)
	No. (%)	No. (%)	No. (%)	No. (%)	No. (%)	No. (%)	No. (%)	No. (%)	No. (%)
Pitting	43 (34)	163 (19)	106 (14)	45 (19)	25 (11)	28 (28)	39 (20)	54 (42.2)	51 (73.9)
Trachyonychia	15 (11)	34 (4)	86 (8)	21 (9)	18 (8)	9 (9)	7 (4)	11 (8.6)	18 (26)
Mottled Lunulae	3 (2)							0	0
Punctate Leukonychia	1 (<1)	17 (2)	21 (3)					16 (12.5)	17 (24.6)
Onychomadesis	2 (1)							0	3 (9.1)
Longitudinal ridging		34 (4)	59 (8)	11 (5)	5 (2)	10 (10)		59 (46.1)	33 (47.8)
Distal notching						2 (2)		23 (18)	30 (43.4)
Lamellar splitting						18 (18)		0	0
Beau's lines						2 (2)		0	0
Ragged cuticles						2 (2)		5 (3.9)	3 (9.1)
Onychorrhexis							3 (1)	0	0
Shiny nail		34 (4)						0	2 (5.6)
Yellow‐brown discoloration		34 (4)						2 (1.6)	3 (9.1)

*Note*: Table used from “Nail changes in alopecia areata: an update and review” by Chelidze and Lipner^12^ (CC BY license).

The results of this study indicated that AA patients with nail abnormalities had more severe disease involvement than patients with no nail changes. In other words, nail changes were observed in 37.7% of the patients with a SALT score of more than 50 and in 29% of the cases with a SALT score of less than 20. The present findings seem to be consistent with other research which found the presence of nail changes in severe forms of AA (AT and AU).^4^, ^9^, ^11^, ^13^, ^26^, ^27^, ^28^, ^29^, ^30^ Garcia‐Hernandez et al.^22^ has proved that the risk of progression to AT and AU is 8.44 times more likely when nail changes are present. Due to a higher proportion of nail changes in AA patients suffering from extensive disease involvement, nail changes have been suggested to be a poor prognostic factor.^12^, ^24^ Furthermore, some studies have indicated that nail abnormalities act as an independent risk factor for treatment‐refractory AA.^10^, ^31^, ^32^, ^33^

The most important clinically relevant finding was the fact that some of the nail changes were significantly associated with disease severity. Distal notching, linear line, and koilonychia had positive associations that could be suggested as poor prognostic factors. On the other hand, yellow‐brown discoloration showed no significant association. Moreover, red spots on the lunula were observed in two cases with universalis pattern that interestingly confirmed the previous reports suggesting this nail abnormality as the most specific sign of severe AA.^2^, ^28^

In our study, AA patients with nail abnormalities were younger (25.31 ± 14.96 years) than those with no nail involvement (32.22 ± 9.77 years). Considering the age groups, nail changes were seen in a large proportion of younger children (less than 10 years) compared to adults (97.1% vs. 76.5%). This is in agreement with Sharma et al.'s^11^ study which reported a higher proportion of nail changes in children than in adults. We also observed more pitting in the pediatric population than in adults as reported in previous studies.^11^, ^31^ Tosti et al.^31^ have indicated that the presence of onychomadesis could be associated with severe AA suggesting a simultaneous injury of both hair and nail matrices; moreover, trachyonychia was observed as an initial symptom of the disease. Similarly, onychomadesis was the specific nail change in the age group under 10 years in our patients and trachyonychia was the other common nail abnormality in this age group, which has been found in almost 60% of the patients with severe AA variants.

In the current study, gender was not associated with nail changes. This result does not support previous findings with some tendencies regarding gender.^2^, ^34^ We have demonstrated that even though the prevalence of nail findings was not significantly different between men and women, some nail findings were more commonly observed between sexes such as brittle nails that were frequent changes in women and trachyonychia and linear line which occurred more significantly in men. Our results were in accordance with Tosti et al.^32^ which found trachyonychia more frequently in men than women.

## 5 LIMITATIONS

One limitation of this study is the small sample size. Second, the evaluation of treatment response regarding nail changes is limited by the use of a cross‐sectional design. Therefore, the current study was not specifically designed to assess the impact of the nail abnormalities on the course of the AA and the response to treatment and also was unable to identify the best drug choice considering the associated nail change. Third, the current study did not perform dermoscopy and nail biopsy. A further study could explore nail changes more precisely using dermoscopy or assess the clinical and pathological associations using nail biopsy.

## 6 CONCLUSION

To our knowledge, this is the first study that not only compared AA patients regarding the presence of nail changes but has also investigated several types of nail changes and their associations with demographic and clinical variables. Previous studies have generally discussed nail abnormalities in AA and assessed their effect on AA progression. Our study revealed that young patients with moderate to severe disease involvement are prone to suffer from nail abnormalities. In addition to pitting, distal notching, and linear line as the most common nail changes, koilonychia, leukonychia, and red spots on the lunula may be poor prognostic predictors for severe AA. Onychomadesis was a rare change exclusively observed in children. Due to the novelty of the current study, future prospective studies with larger study populations using multivariate regression analysis are strongly recommended to identify risk factors for AA‐associated nail changes. Further work needs to be done to establish the poor prognostic nail changes associated with treatment response and also it would be interesting to identify the optimal therapeutic option regarding specific nail changes.

## AUTHOR CONTRIBUTIONS

**Safoura Shakoei**: Conceptualization; methodology. **Golnoosh Seifi**: Writing—original draft; investigation; writing—review & editing. **Farbod Ghanami**: Conceptualization; methodology; writing—original draft. **Narges Ghandi**: Validation; supervision. **Shahin Hamzelou**: Formal analysis; validation. **Maryam Nasimi**: Supervision; validation. **Ifa Etesami**: Validation; supervision; writing—review & editing. All authors have read and approved the final version of the manuscript. Ifa Etesami had full access to all of the data in this study and takes complete responsibility for the integrity of the data and the accuracy of the data analysis.

## CONFLICT OF INTEREST STATEMENT

The authors declare no conflict of interest.

## TRANSPARENCY STATEMENT

The lead author Ifa Etesami affirms that this manuscript is an honest, accurate, and transparent account of the study being reported; that no important aspects of the study have been omitted; and that any discrepancies from the study as planned (and, if relevant, registered) have been explained.

## Supporting information

Supporting information.

## Data Availability

The authors confirm that the data supporting the findings of this study are available within the article and its supplementary materials. The data that support the findings of this study are available from the corresponding author upon reasonable request.
