# Supplementary material for: Clinical and demographic characteristics associated with nail involvement in alopecia areata: A cross‐sectional study of 197 patients
Source: Health Sci Rep. 2024 Apr 1;7(4):e2020. doi: 10.1002/hsr2.2020 (PMC10985222; doi:10.1002/hsr2.2020)
Supplement: Supplementary file 1 — Supporting information. [file HSR2-7-e2020-s001.docx]

Table S.1 Associations between nail abnormalities, gender, disease duration, and AA variants.

| Mottled Lunulae; Beau’s lines; Lamellar splitting; Onychorrhexis; Crumbling | 0 | 0 | 0 | 0 | - | 0 | 0 | 0 | - | 0 | 0 | 0 | 0 | - |
| --- | --- | --- | --- | --- | --- | --- | --- | --- | --- | --- | --- | --- | --- | --- |
| Red spot lunulae | 2 | 1.0 | 0 | 2 (2) | 0.157 | 0 | 1 (1.7) | 1 (1.4) | 0.583 | 0 | 0 | 0 | 2 (3.6) | 0.166 |
| Shiny nail | 2 | 1.0 | 1 (1) | 1 (1) | 0.994 | 2 (2.9) | 0 | 0 | 0.147 | 1 (0.9) | 0 | 0 | 1 (1.8) | 0.884 |
| Onychomadesis | 3 | 1.52 | 3 (3.1) | 0 | 0.079 | 3 (4.4) | 0 | 0 | 0.056 | 2 (1.8) | 0 | 1 (5) | 0 | 0.442 |
| Yellow-brown discoloration | 5 | 2.5 | 4 (4.1) | 1 (1) | 0.171 | 4 (5.9) | 0 | 1 (1.4) | 0.083 | 4 (3.6) | 1 (10) | 0 | 0 | 0.192 |
| Onycholysis | 5 | 2.5 | 1 (1) | 4 (4) | 0.178 | 0 | 1 (1.7) | 4 (5.8) | 0.086 | 2 (1.8) | 0 | 0 | 3 (5.4) | 0.420 |
| Slow growth | 7 | 3.6 | 2 (2) | 5 (5.1) | 0.254 | 4 (5.9) | 2 (3.3) | 1 (1.4) | 0.372 | 3 (2.7) | 0 | 1 (5) | 3 (5.4) | 0.739 |
| Ragged cuticles | 8 | 4.1 | 6 (6.1) | 2 (2) | 0.145 | 4 (5.9) | 3 (5) | 1 (1.4) | 0.382 | 3 (2.7) | 1 (10) | 1 (5) | 3 (5.4) | 0.633 |
| Brittle nails | 10 | 5.1 | 1 (1) | 9(9.1) | 0.01* | 2 (2.9) | 2 (3.3) | 6 (8.7) | 0.235 | 4 (3.6) | 0 | 1 (5) | 5 (8.9) | 0.430 |
| Koilonychias | 11 | 5.6 | 8 (8.2) | 3 (3) | 0.117 | 6 (8.8) | 4 (6.7) | 1 (1.4) | 0.155 | 2 (1.8) | 1 (10) | 4 (20) | 4 (7.1) | 0.009* |
| Disappearing lunula | 14 | 7.1 | 7 (7.1) | 7 (7.1) | 0.984 | 4 (5.9) | 6 (10) | 4 (5.8) | 0.579 | 6 (5.4) | 1 (10) | 0 | 7 (12.5) | 0.203 |
| Trachyonychia | 29 | 14.72 | 22 (22.4) | 7 (7.1) | 0.002* | 16 (23.5) | 4 (6.7) | 9 (13) | 0.024* | 11 (9.9) | 1 (10) | 6 (30) | 11 (19.6) | 0.071 |
| Leukonychia | 35 | 17.75 | 1 (1) | 1 (1) | 0.994 | 0 | 2 (3.3) | 0 | 0.100 | 0 | 1 (10) | 0 | 1 (1.8) | 0.021* |
| Punctate Leukonychia |  |  | 19 (19.4) | 14 (14.1) | 0.324 | 12 (17.6) | 13 (21.7) | 8 (11.6) | 0.302 | 15 (13.5) | 2 (20) | 5 (25) | 11 (19.6) | 0.528 |
| Distal notching | 53 | 26.9 | 34 (34.7) | 19 (19.2) | 0.014* | 23 (33.8) | 19 (31.7) | 11 (15.9) | 0.038* | 20 (18) | 2 (20) | 5 (25) | 26 (46.4) | 0.001* |
| Linear line | 92 | 46.7 | 56 (57.1) | 36 (36.4) | 0.003* | 30 (44.1) | 29 (48.3) | 33 (47.8) | 0.869 | 38 (34.2) | 5 (50) | 10 (50) | 39 (69.6) | 0.000* |
| Pitting | 105 | 53.3 | 54 (55.1) | 51 (51.5) | 0.614 | 41 (60.3) | 36 (60) | 28 (40.6) | 0.032* | 55  (50.5) | 6 (60) | 12 (60) | 31 (55.4) | 0.802 |
| Total nail changes | 374 | 83.8 | 85 (86.7) | 80 (80.8) | 0.259 | 61 (89.7) | 50 (83.3) | 54 (78.3) | 0.191 | 87 (78.4) | 8 (80) | 18 (90) | 52 (92.9) | 0.092 |
|  |  |  | n (%) | |  | n (%) | | |  | n (%) | | | |  |
|  | N | % | M (n=98) | F (n=99) | p-value | ≤3 (n=68) | 3-9 (n=60) | ≥9 (n=69) | p-value | Patchy (n=111) | Ophiasis  (n=10) | AT  (n=20) | AU (n=56) | p-value |
| Nail changes | Frequency | | Gender | | | Disease duration, years | | | | AA variant | | | | |

AA, Alopecia Areata; AT, Alopecia Totalis; AU, Alopecia Universalis; F, female; M, male.

Table S.2 Associations between nail abnormalities, age group and SALT score.

| Nail changes | Age group | | | | SALT score | | | |
| --- | --- | --- | --- | --- | --- | --- | --- | --- |
|  | ≤10 (n=33) | 10-18 (n=36) | >18 (n=128) | p-value | ≤20 (n=66) | 20-50 (n=65) | ≥50 (n=66) | p-value |
|  | n (%) | | |  | n (%) | | |  |
| Total nail changes | 32 (97) | 35 (97.2) | 98 (76.6) | 0.001* | 50 (75.8) | 54 (83.1) | 61 (92.4) | 0.034* |
| Pitting | 22(66.7) | 29(80.6) | 54(42.2) | 0.000* | 26 (39.4) | 43 (66.2) | 38 (54.5) | 0.009* |
| Linear line | 15 (45.5) | 18 (50) | 59(46.1) | 0.906 | 24 (36.4) | 27 (41.5) | 41 (62.1) | 0.007* |
| Distal notching | 17 (51.5) | 13 (36.1) | 23 (18) | 0.000* | 9 (13.6) | 15 (23.1) | 29 (43.9) | 0.000* |
| Leukonychia | 0 | 0 | 2 (1.6) | 0.580 | 0 | 2 (3.1) | 0 | 0.129 |
| Punctate leukonychia | 11 (33.3) | 6 (16.7) | 16 (12.5) | 0.017* | 10 (15.2) | 8 (12.3) | 15(22.7) | 0.255 |
| Trachyonychia | 12 (36.4) | 6 (16.7) | 11 (8.6) | 0.000* | 6 (9.1) | 9 (13.8) | 14 (21.2) | 0.141 |
| Disappearing lunula | 3 (9.1) | 4 (11.1) | 7 (5.5) | 0.451 | 5 (7.6) | 2 (3.1) | 7 (10.6) | 0.241 |
| Koilonychia | 7 (21.2) | 0 | 4 (3.1) | 0.000* | 3 (4.5) | 0 | 8 (12.1) | 0.009* |
| Brittle nails | 1 (3) | 2 (5.6) | 7 (5.5) | 0.842 | 3 (4.5) | 1 (1.5) | 6 (9.1) | 0.140 |
| Ragged cuticles | 3 (9.1) | 0 | 5 (3.9) | 0.159 | 2 (3) | 2 (3.1) | 4 (6.1) | 0.601 |
| Slow growth | 3 (9.1) | 1 (2.8) | 3 (2.3) | 0.168 | 2 (3) | 1 (1.5) | 4 (6.1) | 0.362 |
| Onycholysis | 0 | 0 | 5 (3.9) | 0.251 | 2 (3) | 1 (1.5) | 2 (3) | 0.822 |
| Yellow-brown discoloration | 3 (9.1) | 0 | 2 (1.6) | 0.028* | 2 (3) | 2 (3.1) | 1 (1.5) | 0.811 |
| Onychomadesis | 3 (9.1) | 0 | 0 | 0.001* | 0 | 1 (1.5) | 2 (3) | 0.364 |
| Shiny nail | 0 | 2 (5.6) | 0 | 0.011* | 0 | 0 | 2 (3) | 0.135 |
| Red spot lunulae | 1 (3) | 1 (2.8) | 0 | 0.153 | 0 | 0 | 2 (3) | 0.135 |
| Mottled lunulae; Beau’s lines; Lamellar splitting; Onychorrhexis; Crumbling | 0 | 0 | 0 | - | 0 | 0 | 0 | - |

AA, Alopecia Areata; SALT, Severity of Alopecia Tool
